# A shared respite—The meaning of place for family well-being in families living with chronic illness

**DOI:** 10.3402/qhw.v11.30308

**Published:** 2016-03-07

**Authors:** Liselott Årestedt, Eva Benzein, Carina Persson, Margareta Rämgård

**Affiliations:** 1 Department of Health and Caring Sciences, Faculty of Health and Life Sciences, Linnaeus University, Kalmar, Sweden; 2 Centre for Collaborative Palliative Care, Linnaeus University, Kalmar, Sweden; 3 Department of Care Science, Faculty of Health and Society, Malmö University, Malmö, Sweden

**Keywords:** Family systems nursing, place security, sense of place, family well-being, chronic illness, phenomenological hermeneutics

## Abstract

Living with chronic illness is a family affair that involves ongoing changes and challenges in everyday life. When life changes, the environment is important for family health and well-being. The relation between a place and a family is rarely described, and therefore the aim of this study was to explore the meaning of place for family well-being in families living with chronic illness. A qualitative design was chosen. Data were collected by photovoice combined with narrative family research interviews with 10 families living with chronic illness. A phenomenological hermeneutic analysis was used to interpret the data. The results showed that the meaning of place for family well-being in families living with chronic illness can be described as “a shared respite.” This main theme included three subthemes: “a place for relief,” “a place for reflection,” and “a place for re-creation.” These results were further understood by means of the concept place security. Feeling well means having place security in these families. Through knowledge about the meaning of place for family well-being, health care personnel can stimulate families living with chronic illness to find respite in places that contribute to well-being, both in familiar and new places.

In this study, the focus was on families living with chronic illness and their relation to place. Therefore, Family Systems Nursing theory was combined with concepts from human geography.

Living with chronic illness is not only an individual concern but also a family affair (Årestedt, Persson, & Benzein, 2014; Eggenberger, Meiers, Krumwiede, Bliesmer, & Earle, 2011). In this study “chronic illness” was defined as the irreversible presence, accumulation, or latency of disease states or impairments that involve the total human environment for supportive care and self-care, maintenance of function, and the prevention of further disability (Curtin & Lubkin, 1995). Chronic illness is often described as an unpredictable situation (Markle, Attell, & Treiber, 2015; Röing & Sanner, 2015) in which the body is experienced as weakened and vulnerable. There are often fears about recurrence and sometimes the individual tries to hide symptoms. For the individual person, illness can mean the loss of social network and a strong need for support from close family members (Röing & Sanner, 2015). Chronic illness bring about changes in everyday life with new demands on the individual, (Mahon, O'Brien, & O'Conor, 2014), spouses (Radcliffe, Lowton, & Morgan, 2013), and family functioning (Årestedt et al., 2014). In this situation, a common desire to acknowledge and manage the illness is described (Eggenberger et al., 2011; Eriksson, Asplund, & Svedlund, 2010; Yorgason et al., 2010). Thus, families living with chronic illness have to co-create both a context for living and new patterns in their daily life (Årestedt et al., 2014; Persson & Benzein, 2014).

In families living with chronic illness, family health and well-being are important for how they experience and handle their situation, and vice versa. Family health is a collective experience influenced by values and goals and can be defined as more and something other than the sum of the health of each individual family member. It is about balancing stability, growth, control, and spirituality in response to a changing family environment (Friedman, Bowden, & Jones, 2003). Family members care for one another's well-being (Denham, 2003), and family health is based on the well-being of its members (Hopia, Paavilainen, & Åstedt-Kurki, 2005). In order to maintain family well-being in everyday life, it is important for families to create routines and spend time on something that they value, which creates positive feelings (Ziegert, 2011). Family well-being is about being aware of existing patterns and collaborating to create new ones within the family (Årestedt et al., 2014), and balancing the needs of the individuals and the family with the resources and options available (Kaakinen & Denham, 2014).

The underlying theory in this study is Family Systems Nursing (FSN). From an FSN perspective, the family is the unit of care and nursing interventions focus on interactions, relations, and reciprocity (Benzein, Hagberg, & Saveman, 2008; Wright & Leahey, 2013). In this study, Whall's (1986) definition of family was used: family is a self-defined group of two or more individuals who may, or may not, be bound by blood ties or law, but who function in a way that makes them feel they are a family. Furthermore, in FSN, there is also emphasis on the family as an open system in constant interaction with the environment in which the family functions (Wright & Leahey, 2013). In family nursing literature, the environment is briefly mentioned as an important part of family health. The environmental perspective is mostly described as the culture and the social context, for example, the home, neighborhood, and community (Eddy, Bailey, & Doutrich, 2014; Friedman et al., 2003), and the environment is referred to as parts of families’ micro- and macrosystems (Friedman et al., 2003; Wright & Leahey, 2013). Family nursing mostly describes how family members interact socially but less has been described about the families’ interaction with the material world.

In human geography, it is described that people can develop a relation to a place that gives harmony between body and soul (Casey, 1993; Cresswell, 2004; Holloway & Hubbard, 2001). The concept sense of place is described as an individual experience and refers to the personal and emotional attachment people have to a place. We often have a sense of place about where we live and where we lived during our childhood (Cresswell, 2004; Tuan, 2001). Sense of place is also about human interaction with a specific place, a feeling of close connection and the meaning created (Tuan, 2001). A sense of place emerges from both interactions with the place, as well as social relations at the place. Family relations, traditions, and shared experiences of these places (Kyle & Chick, 2007), as well as memories (Knez, 2006, 2014) are important for developing a sense of place.

In nursing research, sense of place can be related to the concepts of home and at-homeness. Home is described as a place where people can feel safe and live according to their habits (Lindahl, Lidén, & Lindblad, 2011). When living with long-term illness, home can mean a place for self-expression, control, and security (Downing, 2008). This feeling of insideness can change if illness manifestations increase, and the home becomes not only a safe place but also a place of uncertainty about the future, where feelings of homelessness can arise (Bjørn, Ekman, Skott, & Norberg, 2001). The concept at-homeness includesa feeling of being at home and being safe, despite the illness. These feelings are not always connected to the physical home, but are a more existential feeling about being at home (Ohlen, Ekman, Zingmark, Bolmsjo, & Benzein, 2014).

In addition, if a person with illness depends on care outside the home, feeling safe and having meaningful relations to a place are also important. These contribute to a feeling of at-homeness (Browall, Koinberg, Falk, & Wijk, 2013; Crooks & Chouinard, 2006; Falk, Wijk, Persson, & Falk, 2013; Heath, Greenfield, & Redwood, 2015; Moore, Carter, Hunt, & Sheikh, 2013), and a sense of place can be important for the outcome of rehabilitation (Sutton, Rolfe, Landry, Sternberg, & Price, 2012).

Most research about health, illness, and place is performed from an individual perspective and concerns different areas. Carolan, Andrews, and Hodnett (2006) described different aspects of a sense of belonging and how a place can be experienced as a healing place, and thereby contribute to well-being. Andrews (2002) means that the experience of health and place are intertwined and cannot be separated. When combining these, research can tell both patients’ and nurses’ place-related stories and experiences. Carolan et al. (2006) argued that combining concepts about place with nursing science could offer new understandings about the complex relationship between physical place, well-being, and caring. Integrating FSN with concepts of place can generate valuable knowledge of how to support shared family well-being when living with chronic illness.

## Aim

The aim of this study was to illuminate the meaning of place for family well-being in families living with chronic illness.

## Method

### Design

A FSN approach was used for this study and the analysis was based on phenomenological hermeneutics (Lindseth & Norberg, 2004), to uncover the meaning of place for family well-being through interpretation.

### Participants

For the study, the sample of families was recruited deliberately. The criteria for inclusion were; Swedish-speaking families in which an adult member had lived with chronic somatic illness for more than 2 years and at least one family member was invited to an interview. According to Whall (1986), persons with an illness were given information about the study that included a broad definition of family members. In other words, prospective participants were asked to invite someone to whom they felt close. Families with cognitive or speech disorders and persons in palliative care were excluded.

Participants were recruited through patient associations for various illnesses, such as heart and lung illness, neurological illness, diabetes, and rheumatoid arthritis. Participants were also recruited from information provided by nurses and public advertising at a medical clinic in a hospital in the south of Sweden. If family members were interested in participating, they contacted the first author (LÅ) directly or a nurse mediated the contact. Families were given oral and written information about the study. A total of 10 families were recruited. The person with illness had lived with illness between 2 and 42 years. The characteristics of families are shown in Table I.

**Table I:** Family characteristics (*n*=10).

Family members	Person with illness,^a^ age (in years)	Family member (relation to person with illness and age, in years)	Family member (relation to person with illness and age, in years)	Family member (relation to person with illness and age, in years)
Family 1	Woman, 42	Husband, 44	Daughter, 17	Son, 14
Family 2	Woman, 35	Husband, 37	Daughter, 7	Mother, 61 years
Family 3	Man, 35	Wife, 36		
Family 4	Man, 70	Wife, 66	Son, 27	
Family 5	Man, 57	Partner, 40		
Family 6	Man, 82	Wife, 76		
Family 7	Man, 70	Wife 69		
Family 8	Woman, 60	Husband, 59	Daughter, 24	Daughter, 29
Family 9	Woman, 70	Husband, 70	Daughter, 40	
Family 10	Man, 54	Wife, 50		

a The persons with illness were diagnosed with either diabetes, COPD, stroke, renal failure, whiplash, sarcoidosis, Crohn's disease, Parkinson's disease, or rheumatism. All persons with illness received medical and pharmacological treatment due to illness.

### Data collection

Data were collected using photovoice (Hansen-Ketchum & Myrick, 2008; Wang & Burris, 1997) and narrative family interviews (Eggenberger & Nelms, 2007), conducted by the first author (LÅ) between September 2013 and March 2014.

Nine interviews took place in the participants’ homes, and one interview was held at the author's workplace. The interviews lasted 65 to 95 min, and were tape-recorded and transcribed verbatim by the first author (LÅ).

Photovoice (Hansen-Ketchum & Myrick, 2008; Wang & Burris, 1997) is a data-collecting method that combines photos and interviews. Before the family interview, each family was asked to take one or two photos of a context, place, or situation where they, as a family, feel well together. The families took photos of, for example, their own garden, the stable, the kitchen table, the ice skating hall, etc. In order to get to know more about the family and share understandings of family structure, health history and important life events (Meiers, Krumwiede, Denham, & Bell, 2015), each interview started with drawing a genogram, together with the family. A genogram gives a picture over the family constellation and are drawn together with the family. The overview of who is in the family and details about their situation can be used in the beginning of an interview to engage family members in the conversation (Wright & Leahey, 2013). The photos then constitute the starting point for the interview, during which where the family members are asked open and reflective questions: “Can you tell me about the pictures you have taken? In which way do they symbolize your family well-being?” To facilitate an FSN approach, and to obtain variations and co-create stories, family members were asked to reflect on each other's stories. Circular questions (Tomm & Liedén, 2009) and reflective questions are used to obtain deeper descriptions. To enhance trustworthiness, it is important to let all participating family members be heard and to strive for an open atmosphere in which family members feel confident.

### Data analysis

By using phenomenological hermeneutics, the analysis aims to interpret the text through dialectic movements between understanding and explanation (Ricœur, 1976). Ricœur (1976) states that there is a mutual relationship between the phenomenology and hermeneutic philosophy in order to uncover the meaning of lived experiences. The interpretation consists of three phases: naïve understanding, structural analysis, and comprehensive understanding (Lindseth & Norberg, 2004).

In the first step, the text was read and reread several times to grasp the meaning of the text as a whole, and a naïve understanding was formulated. In the second phase, the structural analysis, the text was decontextualized in order to explain the text (Lindseth & Norberg, 2004). A thematic structural analysis was performed, in which the text was divided into meaning units, condensed, abstracted, and organized in subthemes and themes. In this phase, to get an FSN perspective, questions about the text were asked that focused on shared family well-being related to place. Then, the themes and subthemes were reflected upon to validate or invalidate the naïve understanding. Trustworthiness depends on coherence between the parts and the whole (Lindseth & Norberg, 2004), and throughout the analysis, there was a continuous shifting between the individual interview, the whole text, and the interpretation.

In the last phase, the comprehensive understanding, the naïve understanding, and the structural analysis were put together to gain an in-depth interpretation of the recontexualized text (Lindseth & Norberg, 2004). In this phase, the authors’ pre-understanding, the research question, and relevant literature were used to get an understanding of the text as a whole. The authors’ pre-understanding consisted of ideas about shared family well-being and how places can be important to family well-being. In addition, the authors had theoretical knowledge about the concept place and FSN.

### Ethical considerations

Ethical approval was obtained from the Regional Ethical Committee (D-nr: 2013/97-31). In accordance with the Declaration of Helsinki (2008), participants gave written consent after receiving written and oral information about the study. The information included purpose and procedures, the voluntary nature of participation, and the option to withdraw at any time. Confidentiality and secure data storage were guaranteed.

During the interview, the interviewer strived for an open atmosphere and respect for family members’ privacy. This was achieved by striving for sensitivity, and to avoid forcing any person to tell more than either he or she or the family as a unit appeared to want to discuss. The interviewer was also prepared to refer interviewees for professional help if needed.

## Result

### Naive understanding

The meaning of place for well-being in families living with chronic illness is complex and includes many intertwined parts. There is a movement in how a place is experienced and described. A place can be of more or less importance depending on how the illness manifests itself. When illness is more visible it means more talk about illness, more symptoms, more encounters with health care, new pharmacological treatments with possible side effects etc. Due to illness fluctuations, families create and discover new places for their well-being, while the importance of other places decrease in a parallel process.

When living with illness, families appreciate visits to places that bring feelings of well-being.

A place associated with well-being is often a place that the family has visited for many years. Such a place, where various events have taken place, is sometimes connected to childhood memories. Places related to well-being are also often connected to nature, e.g., the sea or a forest. The family home is described as a significant place for well-being and seems to hold great importance, despite being often taken for granted among families. Sometimes, chronic illness forces the family has to spend more time at home, and specific places in the home will stand for well-being.

Places that mean well-being for families are often related to activities that they associate with the place, but it can also be places where the family members can relax, where there are no demands, and they can just do nothing at all.

The fellowship within the family is mentioned as being important, and a specific place gives the family opportunities to be together. There is a great shared effort to feel well despite illness, and everyone in the family strives for this feeling. Families prioritize individual family members’ opportunities to be in a specific place that contributes to well-being, both for the individual and the family.

### Structural analysis

The structural analysis consisted of one main theme and three subthemes. The main theme was “a shared respite” and the subthemes were “a place for relief,” “a place for reflection,” and “a place for re-creation.”

### A shared respite

A place that creates well-being for families living with chronic illness provides a possibility for a shared respite, which facilitates families’ handling the challenges due to illness in daily life. The illness manifests itself in various ways over time, but it is always on the family members’ minds. It then becomes important for them to find a shared respite so they can “recharge their batteries” and find new energy. The place becomes a respite because it is described as harmonious, energizing, and relaxing for families. Metaphors such as “our own little corner of the world,” “a paradise,” and “like another world” were expressed. Many of these specific places also give the individual family member, as well as the family as a unit, a special sense of belonging and a common reference because the family members have spent time in these places for many years. A shared respite means allowing oneself to rest in a secure environment while, at the same time, families reconstruct their roles and relationships to each other. Places also mean great joy for the family.

#### A place for relief

A place that creates shared well-being for families living with chronic illness brings relief to every family member's life situation. In such a place, the illness becomes less visible and may even be improved.

The place also provides a context in which the family is allowed to be themselves. Being yourself in the place means relaxing for a while and not having to answer other people's questions about the illness and how you feel. Great demands are put on families to live or act in a special way in their daily environment when they have to deal with the illness, and they then need to find a place where the illness is less dominating. When being in a place that constitutes well-being, families can relax and focus on the present. These places are described as non-demanding and experienced as a feeling of freedom and connectedness.

One family described how the illness became less visible when they visited their summer cottage:

Wife: He [person with illness] got so well last summer, when we came to the summer cottage. Person with illness: Yes it was last summer, it was a turn for the better, I got a walker, the home health care fixed that, and then I walked, walked home and then walked a bit further, and then I walked home, and finally I could walk without it.

Wife: Yes, and get the mail.

Person with illness: [*crying*] Oh, I become so moved.

Wife: You have to, despite all our sorrows, you have to find positive things, because otherwise you cannot move on.

Person with illness: I don't know what to say, eh … you are happy in a way.

Wife: Yes it′s wonderful.

Person with illness: Everything works so well.

Wife: Yes, it does.

Person with illness: It's hard to describe the feeling, it's like I said before, you feel almost as if you were abroad.

Wife: Yes, it is our oasis. (Family 6)

Families also described specific places in their own home, for example, the sofa, dining area, and patio, where they feel relief, are able to relax and be themselves—all of which contribute to well-being. In these specific places in their homes, everyone knows how the illness usually manifests itself and how to handle these manifestations. Within the family, everyone can be a unique individual without focusing illness. Specific places in the home that contribute to well-being also mean shared activities. Even if not all family members can physically participate in the activities, due to illness, they are connected by a feeling of fellowship. It means relief for families to be together in these specific places at home.

One family described how the patio in their home generates well-being:

Husband: Yes, but when you are round the back of the house, sitting on the patio, it's more ah, you know it is relaxing, it is seldom, when you are around the front it is easy to …

Person with illness: It is expected.

Husband: … that you have to, eh, do things, have to work or something, but the back really is a respite, calm and nice and you feel, it's close to the pool, lying splashing and just having a good time.

Husband: But then if we talk about the illness you might say, when [*person with illness*] feels ill, eh, you can usually still get out, you lie in bed a lot, when you are tired and resting because it is nicer inside, on the sofa, but if the weather makes it OK to go outside you can handle it, there are no tricky stairs or that.

Person with illness: No.

Husband: … and then when you have put everything in its place and all the daily household work is done, you can sit down and take it easy, and you have to do that because it's hard during the periods when she is sick.

Person with illness: Yes.

Mother: Yes.

Husband: Yes, when she has an illness period, then you only have to get everything together, but you need time for respite because otherwise you can′t manage it. (Family 2)

#### A place for reflection

A place that creates well-being also means opportunities for consideration and reflection for families. It is also a place where you can “just be,” where thoughts can be cleared. Life with illness is described as hectic and intense, and requires families to have fixed routines to handle everyday life, for example, to plan for pharmacological treatment and exercise to reduce illness symptoms. In this humdrum and often stressful existence, families need time to reflect and just be in order to feel well. Families described that a place for well-being also meant having time to talk to each other, time they not usually get in their everyday life. Families also described that a place for well-being make them discover new aspects of themselves and each other through the opportunity to relax and just be in a place.

One family described the sofa in their home as a place for reflection:

Husband: You have time to talk.

Person with illness: Yes.

Husband: Yes, really talk [*emphasis*] with each other.

Person with illness: … Friday night and just snuggling in on the sofa and everyone thinks it is cosy in some way as well and then ….

Daughter: Yes, me and mum lying head to foot on the little sofa and the cat lying on top of us.

Person with illness: Yes, it doesn't need to be that grand either, but it could be quite small things.

Husband: Yes.

Person with illness: It is just that you are together and you have time for each other as well. (Family 2)

Individual family members visiting specific places that are related to well-being on their own is associated with just being and giving new energy to him- or herself, as well as to the family as a whole. At times, when illness is more visible, it becomes more important for an individual family member to visit a place that, earlier in life and in other difficult situations, has provided time for reflection and just being.

Even if individual family members visit a place for well-being, one at a time, the place can be well known and shared by the family as a whole, too. Other family members know the place and can describe and attach to it. Sometimes they visit it for individual reflection and just being. In that way, a feeling of well-being can be shared even if only one family member at a time visits the place.

One family member described a place that was well known to the family, as they visited it both individually and together. She said:

Daughter 2: I have a place where I like to go … the seaward wind, if I'm there in the evening I watch the sun go down and if it is in the morning the sun rises and I sit out there and just … And then you feel such peace and you experience how nice everything is and how beautiful it can be.

Person with illness: I went down to the water, alone, just to…

Daughter 1: … clear your thoughts.

Person with illness: Yes, and think and it didn't matter what the weather was like, yes for the well-being, to cope with the situation.

Daughter 2: It feels like you get new energy as well.

Person with illness: Yes.

Daughter: That you recharge your batteries in some way, you can have this feeling and a longing for this feeling to go out and sit there (Family 8)

#### A place for re-creation

When illness becomes more visible, and sometimes makes it impossible for families to visit a specific place that has previously meant well-being to them, a shared re-creation process starts. Families regret the loss of the place and feelings of sadness arise. Families then try to find new ways to re-create the opportunity to be in a place that reminds them of these earlier places.

Memories of a place can constitute the basis for well-being for families when they recall and talk about places from the past. In this process, photos can be helpful in order to remember details about a place. Longing for a place that is not reachable at the moment and planning to be there in the future instills hope in families and contributes to family well-being.

When individual family members have experiences from specific places that have contributed to well-being throughout their lives, it becomes important for them to introduce these places to other family members in real life. These places are often connected to recurrent actions that have provided a form of stabilization throughout the family members’ lives. Because of the illness manifestations, family members have a need to re-create their memories of places that have previously meant well-being for them. Sometimes, the changes in life resulting from living with chronic illness create time and opportunities for family members to visit and discover each other's previous places. By sharing places with each other within the family, well-being is promoted.

One family talked about how they had discovered each other's places due to illness, something that they would otherwise never have done. The partner of the person with illness had cared for horses in stables throughout her life and she described this as “of course, it is the best place in the world, it really is.” The person with illness had never been in a stable before they met. It then became a shared place and he described it as: “Yes the nicest time of the day by far is when you are out there [the stable] and feed your animals, it is completely quiet and the only thing you hear are the birds and them [the horses] chewing and feeling well.” Re-creating this place together, where they felt well in the relaxing atmosphere and with their daily routines together with the horses, were mentioned as a major source of rehabilitation.

## Comprehensive understanding

The meaning of place for family well-being in families living with chronic illness can be described as families needing to be in specific places connected with a feeling of respite, which helps them to handle everyday challenges of illness. These specific places can give rise to emotional feelings for family members and can be related to the theoretical concept sense of place. Even if the sense of place is described as an individual experience (Casey, 1993), the families in this study had developed a shared sense of place. This sense of place is relational if families have an attachment to these places based on earlier experiences. From a systemic point of view, the result can be interpreted as family well-being based not only on social interaction within the family system, but also the families’ interaction with the places. The respite in a specific place consists of relief, reflection, just being, and re-creation, and together they describe the relation between families and places. This deeper connection to place seems to be something more than only an individual or shared emotional feeling of sense of place. Therefore, the concept place security integrated with an FSN perspective was used to interpret the results to wider understanding.

Place security can be defined as a deeper sense of confidence in certain places that follows us through life and includes identity, continuity, and ritualization. Throughout life, we associate situations and relate memories to specific places. When changes in life occur, we often prefer to be in places that represent security to us (Rämgård, 2006, 2009). Social networks, such as family, become important for the feeling of place security (Rämgård, 2006), when places and activities are intertwined and contribute to interaction between people (Rämgård, 2009).

Living with chronic illness is always about handling new information and changes in life. Therefore, it seems important for family members to return to a place where they felt secure before during other changes and difficult situations in life. This points to families’ need for continuity in place over time. They need to return to the same places when they need a respite, and they also re-create and introduce other family members to their previous places. According to Rämgård (2006), people construct their identity through places during childhood and these places become important throughout life. Familiar places contribute to well-being and the results in this study show that families need to share these places with each other and develop a shared security.

This study points out that there are shared activities within a family that contribute to well-being. According to Rämgård (2006), these activities that are related to a specific place can be understood as a form of continuity. They also seem to be a kind of ritual as the same activities are performed over time. Rämgård (2006) has stated that continuity creates a feeling of being at ease even when facing threatening experiences. This takes time to develop, and includes both continuity in the past as well as how the future is envisioned.

This continuity and ritualization of place seems to shape an identity for the family. The core in our personal identity is connected to the physical environment, affecting our self-image and how we understand the world (Rämgård, 2006). The results of this study show that when place and activities are intertwined over time, families often create a shared meaning in these specific places that can be interpreted as a way to develop family identity. According to Denham (2003), family identity has ties to how families collectively interpret memories and meanings of unique attachments to persons, places, and things. New information and experiences from diverse environments can affect family identity, both positively and negatively. If the experience is positive, family well-being can evolve. According to Gregory (2005), personal identity and family identity are connected. To do “normal” and familiar things together can be a way to feel like a family, i.e., strengthen family identity. The results show that a shared respite can facilitate a strengthened family identity.

The results also highlight the fact that families need to be in specific places that are connected with a feeling of respite and relief; it helps them to handle everyday life at times of illness. Many different kinds of places are described, which can be understood as the relation between the family and the place being observed at various geographical levels. The place can be a bigger context, for example, a town, but it can also be a smaller place. The families in this study described the home as being such a small place. There are also specific places in the home that are more related to well-being than others. These places are connected to shared activities within the family that are associated with a “normal” life. This can be related to family health described from a systemic view. It is about family functioning, but also about interactions and exchanges within the family (Friedman et al., 2003). From this perspective, the possibility of being together in a secure place, such as one's home, where the family can feel a respite, can contribute to family well-being. The family's own home can also be connected to family identity and continuity as home often stands for a place where things are familiar and unchanged. Home represents a sense of privacy, security, and safety, and is often related to behavioral consistency, according to the family's routines and rituals (Friedman et al., 2003).

Through family routines and rituals, families can develop a feeling of security through a place. According to Rämgård (2006), place security also has a strong connection to ontological security; when you feel secure in a place, you can also have existential security in life. Social networks, such as family, become important for the feeling of place security. In this study, the families described a changed everyday life due to illness, and they seemed to have a need to be in secure places. Even if the experience of place security is described by Rämgård (2006) as highly individual, this study shows that the concept can also be transferred to a family perspective. This study shows that social relations are intertwined with place, but also how they are connected to each other. Thus, places are an important part of family well-being.

## Methodological considerations

In this study, data were collected on a family level that illuminate the FSN perspective as a way to increase knowledge about family processes (Eggenberger et al., 2011; Eggenberger & Nelms, 2007). In this study, a broad family definition was used, but nevertheless the families consisted of “traditional” family members. Probably, this was a coincidence, depending on whom the persons with illness invited to participate in the interview. In the interview situation, it seemed to be an open climate in which the families spoke freely and were willing to share experiences with each other and the interviewer, even though their stories included new thoughts that they had never talked about within the family. According to Eggenberger and Nelms (2007), participating in family interviews can be a positive experience for the family and they become aware of each other's experiences and thoughts when listening to each other's stories. Starting the interview by doing a genogram together with the family had many benefits. It was a good way for the interviewer to learn more about the family, and was a basis for follow-up questions later in the interview, which drew upon what the family said when doing the genogram. It was also a way to involve and engage all participating family members at the start of the interview, which possibly made it easier for them to talk during the interview.

Photovoice was used to help the families reflect on the meaning of place. It is described as a way to stimulate the interviewees during the interview, and can be used in difficult research areas in order to gain more understanding (Riley & Manias, 2004). Many family members found it helpful to use photos as a starting point as they often had not reflected on places that contribute to well-being. Choosing photos before the interview also seemed to be a way for them to prepare themselves for the interview, and seemed to make it easier for them to focus on shared well-being. Participants described their reflections and discussions when they chose their photos. In the interview situation, they also talked about many places other than the ones chosen in the photos; the images seemed to open up more reflections about other places that contributed to well-being. The authors were aware of that places also can have negative associations, but in this study the focus was on places that means well-being for families living with chronic illness, and families were only asked to tell about places where they feel well together.

In this study, rigor was established by describing the steps of the analysis according to the method by Lindseth and Norberg (2004) and the philosophy of Ricœur (1981). To validate the result and describe a systemic view, quotations was used to strengthen trustworthiness. According to Ricœur (1976), the most relevant interpretation will be described, but it is always possible to argue either for or against an interpretation. The results should therefore be considered as one possible way to understand the meaning of place for family well-being. In order to strengthen credibility, the interpretation was discussed among the co-authors and other researchers, as recommended by Lindseth and Norberg (2004). According to Ricæur (1981), pre-understanding is useful part in the interpretation of the text. In this study, the authors continuously made reflections to be aware of and expand pre-understanding. Reflections were also helpful for the authors to be open-minded about the phenomenon throughout the process.

Transferability was enhanced by a thorough description of the families. The meaning of place for well-being seems to be relevant and important for families, and the result may be transferable to other similar contexts, for example, acute illness or palliative care. These contexts are also examples of difficult and changing situations in life in which people want to be in secure places where, for example, illness is less visible and well-being can be a focus.

## Clinical implications

This study revealed knowledge about the relation between place and family, but also about how place is related to well-being for families living with chronic illness. Through knowledge about families’ experiences of place security, health care personnel can initiate dialogues about places and also promote families to be in places where they feel well and secure.

Health care personnel can encourage families’ re-creation process when illness restricts their lives. In collaboration with family members, health care personnel can help them create and discover new places together by allowing them to talk about memories of previous places where they have felt well.

Health care personnel can also create secure places, for example, at home or at the hospital that fit the unique family's needs according to their experiences of shared well-being. This is important as families living with chronic illness often have repeated encounters with health care services and the place of care becomes a part of life for them.
